# Does Motivation Matter? Analysis of a Randomized Trial of Proactive Outreach to VA Smokers

**DOI:** 10.1007/s11606-016-3687-1

**Published:** 2016-04-12

**Authors:** Elisheva R. Danan, Anne M. Joseph, Scott E. Sherman, Diana J. Burgess, Siamak Noorbaloochi, Barbara Clothier, Sandra J. Japuntich, Brent C. Taylor, Steven S. Fu

**Affiliations:** VA HSR&D Center for Chronic Disease Outcomes Research, Minneapolis VA Health Care System, Minneapolis, MN USA; Department of Medicine, University of Minnesota Medical School, Minneapolis, MN USA; VA New York Harbor Healthcare System, New York City, NY USA; Department of Population Health, New York University School of Medicine, New York City, NY USA; National Center for PTSD, VA Boston Healthcare System, Boston, MA USA; Department of Psychiatry, Boston University School of Medicine, Boston, MA USA

**Keywords:** smoking cessation, motivation, veterans

## Abstract

**Background:**

Current guidelines advise providers to assess smokers’ readiness to quit, then offer cessation therapies to smokers planning to quit and motivational interventions to smokers not planning to quit.

**Objectives:**

We examined the relationship between baseline stage of change (SOC), treatment utilization, and smoking cessation to determine whether the effect of a proactive smoking cessation intervention was dependent on smokers’ level of motivation to quit.

**Design:**

Secondary analysis of a multicenter randomized controlled trial.

**Participants:**

A total of 3006 current smokers, aged 18–80 years, at four Veterans Affairs (VA) medical centers.

Interventions: Proactive care included proactive outreach (mailed invitation followed by telephone outreach), offer of smoking cessation services (telephone or face-to-face), and access to pharmacotherapy. Usual care participants had access to VA smoking cessation services and state telephone quitlines.

**Main Measures:**

Baseline SOC measured with Readiness to Quit Ladder, and 6-month prolonged abstinence self-reported at 1 year.

**Key Results:**

At baseline, 35.8 % of smokers were in preparation, 38.2 % in contemplation, and 26.0 % in precontemplation. The overall interaction between SOC and treatment arm was not statistically significant (p = 0.30). Among smokers in preparation, 21.1 % of proactive care participants achieved 6-month prolonged abstinence, compared to 13.1 % of usual care participants (OR, 1.8 [95 % CI, 1.2–2.6]). Similarly, proactive care increased abstinence among smokers in contemplation (11.0 % vs. 6.5 %; OR, 1.8 [95 % CI, 1.1–2.8]). Smokers in precontemplation quit smoking at similar rates (5.3 % vs. 5.6 %; OR, 0.9 [95 % CI, 0.5–1.9]). Within each stage, uptake of smoking cessation treatments increased with higher SOC and with proactive care as compared with usual care.

**Limitations:**

Mostly male participants limits generalizability. Randomization was not stratified by SOC.

**Conclusions:**

Proactive care increased treatment uptake compared to usual care across all SOC. Proactive care increased smoking cessation among smokers in preparation and contemplation but not in precontemplation. Proactively offering cessation therapies to smokers at all SOC will increase treatment utilization and population-level smoking cessation.

## INTRODUCTION

Current US smokers overwhelmingly want to quit (68.8 %), and most make at least one quit attempt each year (52.4 %), yet they rarely achieve sustained abstinence (6.2 % per year).^1^ As a result, the prevalence of smoking in the US has plateaued at approximately 18 % of adults.^2^ Evidence-based smoking cessation therapies such as medication and counseling significantly increase the success of quit attempts,^3^,^4^ but these therapies are underutilized.^1^,^5^ Current models of care for tobacco cessation treatment rely on highly motivated smokers to initiate therapy (e.g., by calling state telephone quitlines) or on clinical providers to offer therapy. The US Clinical Practice Guideline^6^ instructs providers to offer active therapy only to smokers who are “willing” to quit in the next 30 days. The guideline recommends that smokers who are not ready to quit receive brief motivational interventions (e.g., motivational interviewing) to enhance readiness to quit.

The theoretical justification for evaluating smokers’ readiness to quit prior to offering therapy is rooted in the transtheoretical model (TTM).^7^ This model describes progression through five stages of change (SOC) (precontemplation, contemplation, and preparation for current smokers; action and maintenance for those who have quit) that correlate with ten processes of behavior change. Hundreds of published validation, population, and intervention studies have evaluated the TTM in the context of tobacco use.^8^,^9^ According to the TTM, “action-oriented” interventions such as cessation pharmacotherapy are most effective in the advanced stages.^10^–^12^ Unfortunately, 80 % of U.S. smokers have historically fallen into the precontemplation and contemplation stages.^13^,^14^ As motivated smokers have quit in response to public health campaigns and policy initiatives,^15^ the proportion of smokers in preparation has dropped even further, with levels now at only 9–12 %.^16^,^17^ Efforts to help early-stage smokers transition to higher stages through motivational interviewing have produced mixed results.^18^ As the percentage of smokers in preparation shrinks, the practice of offering active therapy only to those preparing to quit will have diminishing returns.

While the TTM has served as a useful framework for understanding behavior change, it has limitations as the basis for clinical practice guidelines. The TTM systematically underestimates smokers’ motivation to quit,^19^–^21^ as many, if not most, precontemplators and contemplators both want to and try to quit.^22^ In fact, several interventions have documented successful abstinence among precontemplators and contemplators.^23^–^25^ These results substantiate critiques of the construct validity^21^,^26^–^28^ and inherent instability of the TTM stages.^21^,^29^ Interventions that proactively offer evidence-based smoking cessation therapies to all smokers, regardless of SOC, may provide an opportunity to reduce the prevalence of smoking.^30^

The Veterans Victory Over Tobacco Study randomized smokers to usual care or to a proactive, population-based tobacco cessation intervention that offered telephone or in-person counseling, as well as access to cessation medications, to smokers regardless of SOC. The primary results revealed a statistically significant higher population-level 6-month prolonged smoking abstinence rate at 1 year for proactive care (13.5 %) compared with usual care (10.9 %, p = 0.02).^31^ In this secondary analysis, we evaluate the effectiveness of proactive care among smokers at different baseline SOC. Our primary question is whether a proactive outreach intervention will increase prolonged abstinence even among those who say they are not ready to quit. Secondary outcomes include the uptake of cessation therapies and quit attempts by smokers at each SOC.

## METHODS

### Study Design and Participants

The Veterans Victory Over Tobacco Study was a pragmatic randomized controlled trial, and was approved by the institutional review boards of all participating sites. Current smokers (aged 18 to 80 years) were identified using the US Department of Veterans Affairs (VA) electronic medical record health factor data set. Participants were recruited from October 2009 to September 2010 from four VA medical centers (New York, NY; Jackson, MS; Tampa, FL: Minneapolis, MN), and follow-up was completed in November 2011. Additional details of the trial design and methods were described previously.^31^,^32^

### Treatment

The proactive care intervention comprised proactive outreach (mailed materials followed by telephone outreach) combined with an offer of telephone smoking cessation counseling or referral to in-person counseling. Telephone care included a combination of proactive calls from trained counselors at the Minneapolis VA and facilitated access to smoking cessation pharmacotherapy through the participant’s VA provider. The usual care group did not receive proactive outreach but did have access to smoking cessation services through their local VA and their state telephone quitline.

### Data Collection

VA administrative and health care utilization data were obtained from the VA National Patient Care Database. Survey data were collected at baseline and 1-year follow-up.

### Measures

Nicotine dependence was evaluated at baseline and follow-up using time to first morning cigarette and number of cigarettes per day.^33^ SOC was assessed at baseline and follow-up with the ten-point Readiness to Quit Ladder (RQL).^34^ Ladder responses 1 through 10 were categorized into low (1–4), medium (5–6), and high (7–10) levels of readiness that approximate and are referenced hereafter as precontemplation, contemplation, and preparation (adapted from Abrams et al.^34^).

The primary outcome was self-reported 6-month prolonged abstinence at 1-year follow-up, and was assessed among all participants, regardless of treatment utilization. Secondary outcomes included uptake of smoking cessation therapies and quit attempts measured at baseline and follow-up. The use of behavioral counseling (telephone or in-person) and/or smoking cessation medications from any source (including bupropion, varenicline, and nicotine replacement therapy [NRT]) was self-reported. Medications were also assessed using administrative prescription data. Quit attempts were assessed with the question, “During the past 12 months, how many times have you quit smoking intentionally for 24 hours or longer?”

### Analysis

We used stratified random sampling (by site) to select the study sample and a completely randomized block design to assign participants to the intervention or usual care. Accordingly, our estimations, testing, and modeling procedures are stratified analyses. To compare baseline characteristics across the SOC, the weighted stratified Wald χ^2^ was used for categorical variables and the weighted stratified *F*-test was used for continuous variables. The weights were inverses of the sampling proportions from each site. To account for possible intra-block correlations, logistic regression mixed modeling was used to test the effect of SOC on the primary outcome, 6-month prolonged abstinence. All models included the intervention and blocking factor (site). We tested the interaction between SOC and treatment arm with respect to the primary outcome. Randomization was not stratified by SOC, which allowed for potentially imbalanced covariates (both measured and unmeasured) between the two treatment arms within each SOC to occur by chance, and this may have created a biased interaction term. To control for between-group imbalances, we have presented a stratified analysis, comparing the treatment effect within each SOC separately. Imbalanced characteristics at the 0.05 significance level were included in the models as adjusting covariates.

To handle non-response, we hypothesized that this might depend on the unobserved smoking status of the subject; that is, we assumed a not-missing-at-random (NMAR) mechanism. We modeled the joint distribution of abstinence status and response status for the logistic regressions using an expectation–maximization algorithm to find maximum likelihood estimators, as described by Ibrahim and colleagues.^35^,^36^ This likelihood-based NMAR method creates two data sets, one that assumes all non-responders are smokers, and another that assumes they are all quitters. Then, through a series of iterative weightings, it produces maximum likelihood estimates. The data analysis for this paper including the macro for likelihood-based NMAR modeling was generated using SAS/STAT software, version 9.2 (SAS Institute Inc., Cary, NC, USA).

## RESULTS

Of the 5123 eligible, randomized participants, 3006 provided complete baseline survey data, including the RQL, and thus constitute the sample for this secondary analysis (58.5 % [1473/2519] of those randomized to the proactive care intervention and 58.9 % [1533/2604] of those in usual care). At baseline, 781 smokers were in precontemplation (26.0 %), 1148 were in contemplation (38.2 %), and 1077 were in preparation (35.8 %) (Table 1).

**Table 1:** Participant Characteristics by Baseline Stage of Change

	Total, *n*	Precontemplation, *n* (%)*or mean (SD)	Contemplation, *n* (%) or mean (SD)	Preparation, *n* (%) or mean (SD)	p value
All participants	3006	781	1148	1077	–
Treatment group					
Usual care	1533 (51.3)	396 (50.6)	585 (50.7)	552 (52.5)	0.64
Proactive care	1473 (48.7)	385 (49.5)	563 (49.2)	525 (47.5)	
Demographic characteristics:					
Age (years)	57.7 (10.6)	59.4 (10.3)	57.0 (10.7)	57.3 (10.5)	<0.001
Race					
White	1852 (67.2)	543 (74.4)	737 (70.0)	572 (58.4)	<0.001
Black	774 (22.2)	154 (16.4)	280 (20.2)	340 (29.0)	
Hispanic	85 (4.5)	41 (4.2)	59 (3.6)	85 (5.8)	
Other	95 (6.1)	43 (5.1)	72 (6.2)	80 (6.8)	
Gender					
Male	2838 (94.4)	741 (94.9)	1080 (94.2)	1017 (94.1)	0.71
Marital status					
Married	1460 (50.0)	337 (44.8)	602 (53.6)	521 (50.0)	0.001
Socioeconomic status:					
Income ($)					
< 10,000	511 (17.0)	123 (16.1)	185 (16.0)	203 (18.9)	0.001
10,000–20,000	879 (30.8)	238 (32.8)	307 (28.1)	334 (32.4)	
20,001–40,000	841 (30.5)	841 (29.7)	207 (30.4)	323 (31.3)	
≥ 40,001	593 (21.6)	155 (21.4)	266 (25.5)	172 (17.4)	
Social and environmental pressures:					
Home smoking rules					
Not allowed anywhere	1116 (41.1)	237 (33.3)	406 (39.8)	473 (48.6)	<0.001
Allowed some places/times	602 (20.7)	142 (19.2)	219 (20.0)	241 (22.3)	
Allowed anywhere	1087 (38.2)	354 (47.5)	438 (40.2)	295 (28.7)	
Friends who smoke					
None	454 (16.2)	116 (15.8)	171 (16.2)	167 (16.5)	0.001
< half	809 (28.7)	185 (24.6)	296 (27.9)	328 (32.7)	
About half	614 (22.2)	163 (22.3)	238 (22.7)	213 (21.7)	
> half	530 (19.4)	134 (18.8)	217 (21.0)	179 (18.0)	
All	384 (13.6)	130 (18.6)	135 (12.2)	119 (11.2)	
People important to me want me to quit smoking					
Strongly disagree to neutral	580 (21.6)	267 (37.8)	188 (17.9)	125 (13.2)	<0.001
Somewhat agree	628 (22.8)	196 (26.7)	234 (23.1)	198 (19.3)	
Strongly agree	1569 (55.6)	263 (35.5)	635 (59.0)	671 (67.5)	
Smoking behaviors:					
Cigarettes per day					
≤ 10	1018 (31.3)	211 (24.7)	290 (22.3)	517 (46.7)	<0.001
11–20	1286 (45.0)	341 (46.3)	547 (49.5)	398 (38.9)	
≥ 21	652 (23.7)	213 (29.0)	301 (28.3)	138 (14.4)	
Time to first cigarette, min					
≥ 31	809 (25.8)	185 (23.2)	249 (20.7)	375 (33.6)	<0.001
6–30	1535 (52.6)	402 (52.2)	615 (55.1)	518 (50.1)	
< 5	638 (21.6)	188 (24.6)	278 (24.2)	172 (16.3)	
Quit in past 12 months					
Yes	1673 (54.4)	181 (22.4)	626 (53.2)	866 (80.6)	<0.001
Longest quit length					
Never quit	255 (8.3)	128 (16.3)	72 (6.1)	55 (4.7)	<0.001
< 1 month	824 (27.1)	230 (29.5)	334 (27.9)	260 (24.2)	
1–6 months	809 (27.2)	187 (24.4)	309 (27.5)	313 (28.9)	
> 6 months	1092 (37.5)	225 (29.8)	426 (38.5)	441 (42.2)	

* Observed count (weighted column proportion)

Within precontemplation and contemplation, observed baseline characteristics were balanced across treatment groups (Tables 3 and 4, available online). However, for the preparation group, male gender and level of agreement with the statement “People important to me want me to quit smoking” were not balanced across treatment groups (Table 5, available online). These imbalanced variables were included in the complete case and the NMAR models as adjusting covariates.

### Primary Outcome: 6-Month Prolonged Abstinence (Table 2)

**Table 2:** Primary Outcome: 6-Month Prolonged Abstinence by Baseline Stage of Change and Treatment Arm

Baseline stage of change	Participants, n (%)*		Odds ratio^†^ (95 % CI) Complete case model^‡^ *n* = 2351	Odds ratio^†^ (95 % CI) NMAR model^§^ *n* = 3006
	Usual care, *n* = 1236	Proactive care, *n* = 1115		
Precontemplation	18 (5.6 %)	15 (5.3 %)	0.9 (0.5, 1.9)	1.2 (0.8, 1.8)
Contemplation	31 (6.5 %)	48 (11.0 %)	**1.8** (1.1, 2.8)	**1.8** (1.3, 2.4)
Preparation	60 (13.1 %)	82 (21.1 %)	**1.8** (1.2, 2.6)	**1.6** (1.4, 2.0)

* Observed count (weighted column proportion)

† Usual care is the reference group

‡ Among follow-up survey respondents

^§^ Likelihood-based NMAR (not-missing-at-random) model accounting for non-response

Six-month prolonged abstinence at 1 year varied by baseline SOC (5.4 % for precontemplators, 8.6 % for contemplators, and 17.1 % for preparers [p < 0.001]). The overall interaction between SOC and treatment arm was not statistically significant (p = 0.30). Among smokers in preparation, those randomized to the proactive intervention were more likely to quit than those in usual care (21.1 % vs 13.1 %, respectively, *p* = 0.003). Logistic regression mixed modeling analysis, taking into account treatment arm and facility as well as the adjusting covariates described above, found a significant effect of proactive care compared with usual care among preparers (OR, 1.8 [95 % CI, 1.2–2.6]). Similarly, smokers in contemplation who were randomized to the proactive intervention were more likely to quit than those in usual care (11.0 % vs 6.5 %, p = 0.018; OR, 1.8, [95 % CI, 1.1–2.8]).). Smokers in precontemplation quit at similar rates in the two treatment arms (5.3 % vs 5.6 %, p = 0.85; OR, 0.9, [95 % CI, 0.5–1.9]). Analyses accounting for nonresponse using likelihood-based NMAR models showed a similar effect of the proactive care intervention on prolonged abstinence at each SOC (Table 2). Of the 254 study participants who achieved 6-month prolonged abstinence, 55.6 % began the study in preparation, while 44.4 % were not ready to quit at baseline (12.5 % began in precontemplation and 31.9 % in contemplation).

### Secondary Outcomes

#### Uptake of Smoking Cessation Therapies (Fig. 1)

**Figure 1:**
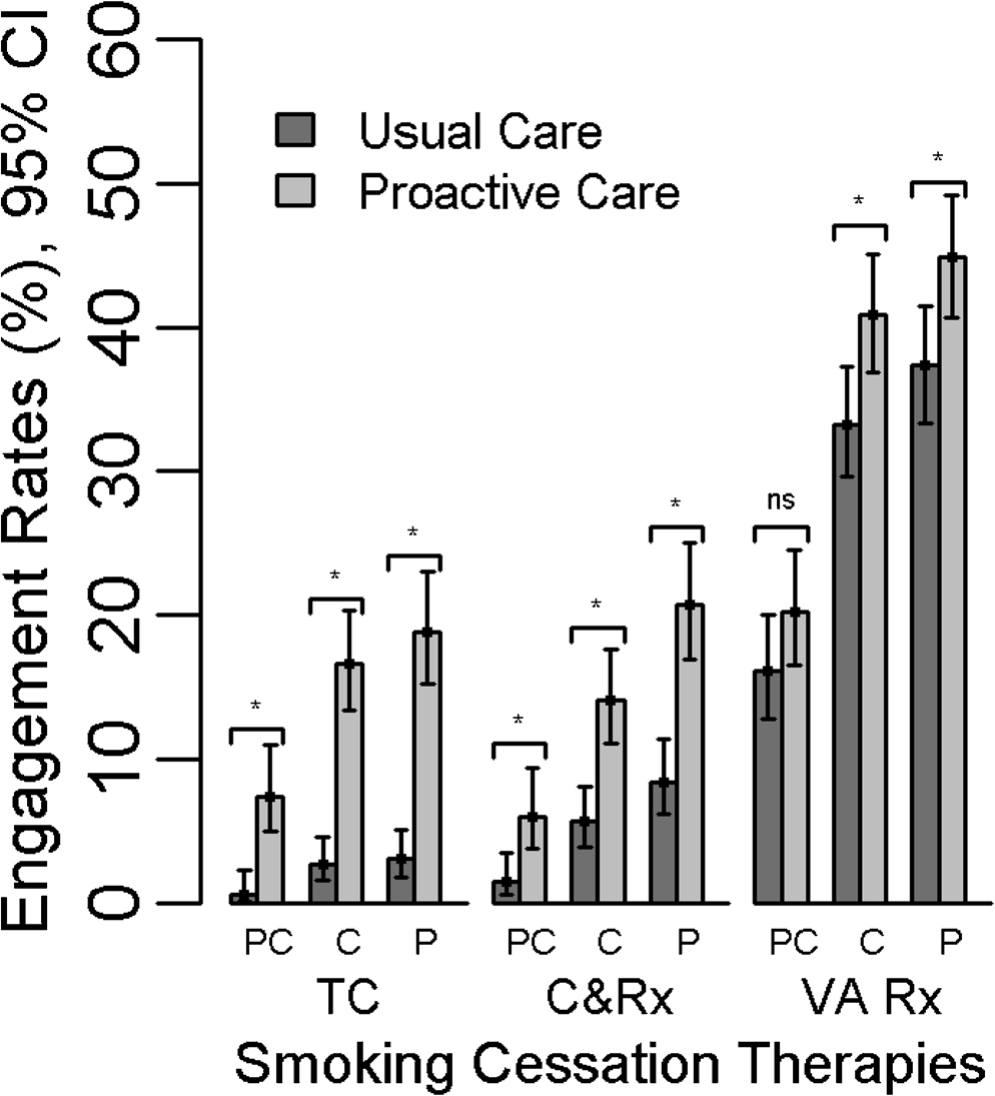
Engagement with smoking cessation therapies by baseline stage of change and treatment arm. PC = precontemplation, C = contemplation, P = preparation, ns = not significant, TC = telephone counseling, C&Rx = counseling & medication, VA Rx = VA-prescribed medication. * 95 % confidence interval for odds ratio does not contain 1.

Smokers at all SOC who were randomized to proactive care engaged in telephone counseling at significantly higher rates than usual-care smokers (7.4 vs. 0.6 % for precontemplation; 16.6 vs 2.7 % for contemplation; 18.8 vs. 3.1 % for preparation) and reported combined therapy with counseling and cessation medication at significantly higher rates than usual care smokers (6.0 vs. 1.5 % for precontemplation; 14.1 vs. 5.7 % for contemplation; 20.7 vs 8.4 % for preparation). Smokers in all SOC who received proactive care were more likely than usual care participants to be prescribed cessation medications through the VA, though this difference reached statistical significance only for contemplation and preparation (20.2 vs. 16.1 % for precontemplation [NS]; 40.9 vs. 33.3 % for contemplation; 44.9 vs. 37.4 % for preparation). Study participants reported using in-person counseling or attending VA smoking cessation clinics at low rates that were not significantly different by study arm or by baseline SOC.

#### Quit Attempts

Baseline SOC predicted the likelihood of making a quit attempt during the study period. Overall, 31.3 % of precontemplators, 55.6 % of contemplators, and 73.5 % of preparers reported making at least one 24-hour quit attempt during the study follow-up period. There was no significant difference in quit attempts between proactive and usual care.

## DISCUSSION

This proactive population-based smoking cessation intervention included smokers at all stages of change (SOC), and increased long-term prolonged abstinence for smokers in both preparation and contemplation. Smokers in precontemplation did not quit at higher rates in response to proactive outreach, but they were more likely to try evidence-based cessation interventions, including telephone counseling and combined counseling and pharmacotherapy. Proactively offering evidence-based cessation therapies to all smokers led to increased therapeutic engagement and higher long-term population-level quit rates.

Our assessment of an overall interaction between SOC and treatment group revealed no statistically significant difference among SOC subgroups with respect to the intervention. Interaction tests are often underpowered and may be biased when the study is not designed to detect these subgroup effects and randomization is not stratified by subgroup. Thus, we presented additional stratified analyses demonstrating the effect of proactive outreach on smokers at each baseline stage. Among highly motivated smokers (preparation), 73.5 % of whom made at least one quit attempt during follow-up, proactive outreach increased the likelihood of quitting successfully by 50 % (21.1 % vs 13.1 %). Though proactive outreach targets less motivated smokers,^37^ we found that even highly motivated smokers benefited from proactive outreach through increased uptake of cessation therapies.

Proactive outreach to contemplators appeared to increase cessation by helping smokers overcome high nicotine dependence through the use of evidence-based therapies. Contemplators had attempted to quit in the past at rates similar to preparers, but had much higher levels of nicotine dependence (Table 1). A history of past quit attempts is a predictor of future attempts, but nicotine dependence levels predict the success of those attempts.^38^,^39^ Increased therapeutic engagement by contemplators resulted in more successful quit attempts. Our finding that treatment engagement was associated with cessation (OR 1.55 [95 % CI 1.06–2.28] for use of combined therapy) supports this proposed mechanism.

Although proactive outreach increased treatment engagement among precontemplators, we did not observe a difference in smoking cessation rates. It may be that proactive outreach that offers standard cessation therapies is ineffective for precontemplators, who face greater barriers to quitting^40^ and may require tailored or high-intensity therapy.^23^ Alternatively, our analysis may have been underpowered to show a difference in cessation among precontemplators, given the smaller size of the subgroup and lower baseline quit rates. Further research is needed for a definitive answer to this question. We found no evidence that the proactive intervention increased unsuccessful quit attempts among precontemplators (31.4 % of precontemplators in usual care and 31.3 % in proactive care made at least one quit attempt during follow-up [*p* = 0.98]), and thus have no reason to believe that proactive care poses harm to precontemplators.

Our results replicate and extend prior research that has evaluated population-based smoking cessation interventions for smokers at all SOC. Since 1995, when Curry and colleagues first demonstrated the potential effectiveness of telephone-based interventions with non-volunteer smokers at all SOC,^25^ dozens of telephone-based trials in various populations have supported this proactive approach. However, most have measured only point-prevalent abstinence and/or found short-term (6–9-month follow-up) effects.^37^,^41^,^42^ In contrast, our proactive care intervention achieved prolonged abstinence at long-term (12-month) follow-up among both preparers and contemplators. One reason for this robust effect may be that we combined population-based outreach (using electronic health technology to identify current smokers and to offer telephone-based care) with individual care management (linking care to VA providers to facilitate pharmacotherapy). Smokers in proactive care were much more likely to use combined counseling and pharmacotherapy (Fig. 1), which has been shown to be highly effective.^4^,^6^

While earlier telephone counseling studies have included smokers at all SOC, those studies did not provide information as to whether smokers at lower SOC benefitted from the interventions or merely diluted the population-level treatment effect. In 2015, Haas and colleagues^43^ addressed this deficiency, reporting that their telephone-based intervention using interactive voice response increased abstinence both among those planning to quit within the next 30 days and among those with no plans to quit. Our report provides additional information on the magnitude of the treatment effect on less motivated smokers and divides less motivated smokers into subgroups of precontemplators and contemplators.

Interest in treating smokers at all SOC has grown over the past decade. In 2005, Pisinger and colleagues first reported the success of the Inter99 trial, which found that a high-intensity intervention could engage less motivated smokers and increase rates of abstinence.^23^,^44^ Several editorials, citing the success of Inter99, have questioned current guideline recommendations to assess motivation to quit prior to offering cessation therapy.^26^,^45^ Aveyard and colleagues conducted a systematic review and meta-analysis of brief physician interventions among smokers at all motivation levels and concluded that combining physician advice to quit with the offer of cessation support motivated an additional 40–60 % of smokers to attempt to quit, compared with advice alone.^30^ The authors suggested that offering assistance in quitting to smokers at all SOC may be effective because the offer itself increases confidence in success. To explain why less motivated smokers often respond to offers of cessation therapy, others have advanced an alternative theory of smoking cessation based in “catastrophe theory,” which posits that motivational tension fluctuates, and small triggers can induce apparently spontaneous quit attempts.^46^,^47^

Carpenter and colleagues subsequently reported that among unmotivated smokers, NRT sampling was more successful for inducing quit attempts and short-term cessation than practice quit attempts alone.^48^ Two additional small studies compared the offer of NRT to usual care and found promising short-term results regardless of motivation.^49^,^50^ After more than a decade of small studies, meta-analyses, and editorials, we now report on the largest low-intensity, pragmatic, proactive care intervention with long-term outcomes among smokers who were not planning to quit at baseline.

Limitations include a study population of mostly older male US veteran smokers, which may limit generalizability to other populations of smokers. Our study, a non-pre-specified subgroup analysis, is restricted to those participants who completed the baseline survey questions that established SOC, and thus this sample may be more engaged than the overall population. Follow-up data availability is limited to an even smaller group. We address the potential for response bias in follow-up data by including a likelihood-based NMAR model analysis. In the primary analysis,^31^ we also addressed possible differential non-response between treatment groups, and found that taking into account non-response bias did not substantially alter the results. Additional limitations result from inconsistencies in operationalizing the TTM across the literature, making it difficult to compare our SOC with those in other studies.^9^

This large, pragmatic randomized trial of a telephone-based intervention demonstrated increased uptake of smoking cessation therapies and prolonged abstinence at 1 year both for smokers who were already planning to quit and for those who were not. Similar to the results of prior studies,^23^,^25^ we found that among participants who quit successfully, nearly half began the study stating that they were not ready to quit. Restricting therapy to only those in the preparation stage would exclude 64 % of the smokers in our sample, and 44 % of those who quit. Our results add to the growing body of evidence that smoking cessation therapy should be proactively offered to all smokers, regardless of stated plans to quit.

## Appendix 1

(Table 3).

**Table 3:** Baseline Participant Characteristics by Treatment Group Within Precontemplation

	Total, *n*	Proactive care, *n* (%)^*^ or mean (SD)	Usual care, *n* (%) or mean (SD)	p value
All participants	781	385	396	–
Demographic characteristics:				
Age (years)	59.4 (10.3)	58.8 (10.6)	59.9 (10.0)	0.06
Race				
White	543 (74.4)	256 (71.7)	287 (77.0)	0.25
Black	154 (16.4)	84 (17.6)	70 (15.2)	
Hispanic	41 (4.2)	20 (4.3)	21 (4.0)	
Other	43 (5.1)	25 (6.2)	18 (3.8)	
Gender				
Male	741 (94.9)	366 (95.2)	375 (94.6)	0.71
Marital status				
Married	337 (44.8)	181 (48.3)	156 (41.3)	0.06
Socioeconomic status:				
Income, ($)				
< 10,000	123 (16.1)	64 (17.3)	59 (15.01)	0.43
10,000–20,000	238 (32.8)	103 (29.8)	135 (35.6)	
20,001–40,000	841 (29.7)	107 (31.3)	100 (28.2)	
≥ 40,001	155 (21.4)	76 (21.7)	79 (21.2)	
Social and environmental pressures:				
Home smoking rules				
Not allowed anywhere	237 (33.3)	121 (35.1)	116 (31.5)	0.20
Allowed some places/times	142 (19.2)	73 (20.8)	69 (17.7)	
Allowed anywhere	354 (47.5)	161 (44.1)	193 (50.8)	
Friends who smoke				
None	116 (15.8)	51 (14.4)	65 (17.0)	0.75
< half	185 (24.6)	91 (24.6)	94 (24.6)	
About half	163 (22.3)	79 (21.4)	84 (23.1)	
> half	134 (18.8)	70 (20.4)	64 (17.2)	
All	130 (18.6)	64 (19.2)	66 (18.1)	
People important to me want me to quit smoking				
Strongly disagree to neutral	267 (37.8)	123 (36.4)	144 (39.1)	0.19
Somewhat agree	196 (26.7)	109 (29.9)	87 (23.7)	
Strongly agree	263 (35.5)	118 (33.7)	145 (37.2)	
Smoking behaviors:				
Cigarettes per day				
≤ 10	211 (24.7)	111 (26.4)	100 (23.1)	0.35
11–20	341 (46.3)	157 (43.7)	184 (48.9)	
≥ 21	213 (29.0)	107 (30.0)	106 (28.0)	
Time to first cigarette (min)				
≥ 31	185 (23.2)	94 (23.4)	91 (23.0)	0.95
6–30	402 (52.2)	196 (51.6)	206 (52.7)	
< 5	188 (24.6)	92 (25.0)	96 (24.3)	
Quit in past 12 months				
Yes	181 (22.4)	91 (22.8)	90 (22.1)	0.80
Longest quit length				
Never quit	128 (16.3)	65 (16.8)	63 (15.8)	0.25
< 1 month	230 (29.5)	126 (32.6)	104 (26.4)	
1–6 months	187 (24.4)	83 (22.5)	104 (26.3)	
> 6 months	225 (29.8)	107 (28.1)	118 (31.5)	

* Observed count (weighted column proportion)

## Appendix 2

**Table 4:** Baseline Participant Characteristics by Treatment Group Within Contemplation

	Total, *n*	Proactive care, *n* (%)^*^ or mean (SD)	Usual care, *n* (%) or mean (SD)	p value
All participants	1148	563	585	–
Demographic characteristics:				
Age (years)	57.0 (10.7)	56.6 (10.9)	57.2 (10.4)	0.32
Race				
White	737 (70.0)	358 (69.5)	287 (70.5)	0.18
Black	280 (20.2)	150 (22.2)	130 (18.3)	
Hispanic	59 (3.6)	26 (3.0)	33 (4.1)	
Other	72 (6.2)	29 (5.3)	43 (7.1)	
Gender				
Male	1080 (94.2)	530 (94.3)	550 (94.1)	0.88
Marital status				
Married	602 (53.6)	292 (52.8)	310 (54.4)	0.60
Socioeconomic status:				
Income ($)				
< 10,000	185 (16.0)	85 (15.1)	100 (16.8)	0.81
10,000–20,000	307 (28.1)	154 (28.2)	153 (28.0)	
20,001–40,000	207 (30.4)	164 (31.6)	159 (29.3)	
≥ 40,001	266 (25.5)	132 (25.2)	134 (25.9)	
Social and environmental pressures:				
Home smoking rules				
Not allowed anywhere	406 (39.8)	209 (41.4)	197 (38.3)	0.45
Allowed some places/times	219 (20.0)	97 (18.5)	122 (21.4)	
Allowed anywhere	438 (40.2)	214 (40.1)	224 (40.3)	
Friends who smoke				
None	171 (16.2)	88 (17.1)	83 (15.2)	0.26
< half	296 (27.9)	132 (25.1)	164 (30.5)	
About half	238 (22.7)	117 (22.4)	121 (23.1)	
> half	217 (21.0)	109 (21.7)	108 (20.4)	
All	135 (12.2)	71 (13.7)	64 (10.8)	
People important to me want me to quit smoking				
Strongly disagree to neutral	188 (17.9)	95 (18.3)	93 (17.5)	0.30
Somewhat agree	234 (23.1)	102 (21.0)	132 (25.2)	
Strongly agree	635 (59.0)	321 (60.7)	314 (57.4)	
Smoking behaviors:				
Cigarettes per day				
≤ 10	290 (22.3)	140 (22.1)	150 (22.4)	0.94
11–20	547 (49.5)	274 (50.0)	273 (48.9)	
≥ 21	301 (28.3)	143 (27.9)	158 (28.6)	
Time to first cigarette (min)				
≥ 31	249 (20.7)	116 (20.0)	133 (21.4)	0.41
6–30	615 (55.1)	298 (54.1)	317 (56.1)	
< 5	278 (24.2)	146 (26.0)	132 (22.5)	
Quit in past 12 months				
Yes	626 (53.2)	307 (54.4)	319 (52.1)	0.46
Longest quit length				
Never quit	72 (6.1)	38 (6.6)	34 (5.5)	0.70
< 1 month	334 (27.9)	172 (29.1)	162 (26.8)	
1–6 months	309 (27.5)	149 (27.1)	160 (27.9)	
> 6 months	426 (38.5)	201 (37.3)	225 (39.7)	

* Observed count (weighted column proportion)

## Appendix 3

**Table 5:** Baseline Participant Characteristics by Treatment Group Within Preparation

	Total, *n*	Proactive care, *n* (%)^*^ or mean (SD)	Usual care, *n* (%) or mean (SD)	p value
All participants	1077	525	552	–
Demographic characteristics:				
Age (years)	57.4 (10.5)	57.2 (10.3)	57.6 (10.8)	0.47
Race				
White	572 (58.4)	260 (55.0)	312 (61.6)	0.20
Black	340 (29.0)	178 (31.2)	162 (27.1)	
Hispanic	85 (5.8)	43 (6.2)	42 (5.4)	
Other	80 (6.8)	44 (7.7)	36 (5.9)	
Gender				
Male	1017 (94.1)	504 (95.7)	513 (92.6)	0.045
Marital status				
Married	521 (50.0)	250 (50.0)	271 (50.1)	0.98
Socioeconomic status:				
Income ($)				
< 10,000	203 (18.9)	98 (19.0)	105 (18.9)	0.91
10,000–20,000	334 (32.4)	170 (33.4)	164 (31.6)	
20,001–40,000	311 (31.3)	146 (31.2)	165 (31.4)	
≥ 40,001	172 (17.4)	81 (16.5)	91 (18.1)	
Social and environmental pressures:				
Home smoking rules				
Not allowed anywhere	473 (48.6)	227 (48.3)	246 (48.9)	0.58
Allowed some places/times	241 (22.3)	113 (21.7)	128 (23.7)	
Allowed anywhere	295 (28.7)	151 (30.1)	144 (27.4)	
Friends who smoke				
None	167 (16.5)	85 (16.7)	82 (16.3)	0.96
< half	328 (32.7)	149 (31.5)	179 (33.7)	
About half	213 (21.7)	104 (22.0)	109 (21.5)	
> half	179 (18.0)	92 (18.8)	87 (17.3)	
All	119 (11.2)	59 (11.1)	60 (11.2)	
People important to me want me to quit smoking				
Strongly disagree to neutral	125 (13.2)	47 (10.1)	78 (16.0)	0.031
Somewhat agree	198 (19.3)	99 (19.7)	99 (18.9)	
Strongly agree	671 (67.5)	332 (70.3)	339 (65.1)	
Smoking behaviors:				
Cigarettes per day				
≤ 10	517 (46.7)	244 (45.3)	273 (48.0)	0.67
11–20	398 (38.9)	201 (40.4)	197 (37.7)	
≥ 21	138 (14.4)	67 (14.4)	71 (14.4)	
Time to first cigarette, min				
≥ 31	375 (33.6)	172 (32.5)	203 (34.5)	0.75
6–30	518 (50.1)	259 (50.5)	259 (49.8)	
< 5	172 (16.3)	88 (17.0)	84 (15.7)	
Quit in past 12 months				
Yes	866 (80.6)	414 (80.0)	452 (81.1)	0.66
Longest quit length				
Never quit	55 (4.7)	30 (5.5)	25 (4.1)	0.11
< 1 month	260 (24.2)	140 (26.4)	120 (22.2)	
1–6 months	313 (28.9)	155 (29.8)	158 (28.2)	
> 6 months	441 (42.2)	194 (38.4)	247 (45.6)	

* Observed count (weighted column proportion)
